# Mechanisms of Dyslipoproteinemias in Systemic Lupus Erythematosus

**DOI:** 10.1080/17402520600876945

**Published:** 2006

**Authors:** Eduardo F. Borba, Jozelio F. Carvalho, Eloísa Bonfá

**Affiliations:** Rheumatology Division São Paulo University Medical School Hospital São Paulo SP Brazil

## Abstract

Autoimmunity and inflammation are associated with marked changes in lipid and lipoprotein metabolism in SLE. Autoantibodies and cytokines are able to modulate lipoprotein lipase (LPL) activity, a key enzyme in lipid metabolism, with a consequent “lupus pattern” of dyslipoproteinemia characterized by elevated levels of very low-density lipoprotein cholesterol (VLDL) and triglycerides (TG) and lower high-density lipoprotein cholesterol (HDL) levels. This pattern favors an enhanced LDL oxidation with a subsequent deleterious foam cell formation. Autoantibodies and immunocomplexes may aggravate this oxidative injury by inducing accumulation and deposition of oxLDL in endothelial cells. Drugs and associated diseases usually magnify the close interaction of these factors and further promote the proatherogenic environment of this disease.


*Clinical & Developmental Immunology*, June–December 2006; 13(2–4): 203–208

Taylor & Francis

Taylor & Francis Group

### Mechanisms of dyslipoproteinemias in systemic lupus erythematosus

EDUARDO F. BORBA, JOZELIO F. CARVALHO, & ELOÍSA BONFÁ

*Rheumatology Division, São Paulo University Medical School Hospital, São Paulo, SP, Brazil*

**Abstract**
Autoimmunity and inflammation are associated with marked changes in lipid and lipoprotein metabolism in SLE. Autoantibodies and cytokines are able to modulate lipoprotein lipase (LPL) activity, a key enzyme in lipid metabolism, with a consequent "lupus pattern" of dyslipoproteinemia characterized by elevated levels of very low-density lipoprotein cholesterol (VLDL) and triglycerides (TG) and lower high-density lipoprotein cholesterol (HDL) levels. This pattern favors an enhanced LDL oxidation with a subsequent deleterious foam cell formation. Autoantibodies and immunocomplexes may aggravate this oxidative injury by inducing accumulation and deposition of oxLDL in endothelial cells. Drugs and associated diseases usually magnify the close interaction of these factors and further promote the proatherogenic environment of this disease.

**Keywords:** *Systemic lupus erythematosus, lipoprotein, dyslipoproteinemia, hyperlipidemia, atherosclerosis, inflammation*

### Introduction

Atherosclerosis has been increasingly recognized as an important cause of morbidity and mortality in systemic lupus erythematosus (SLE) (Hahn 2003; Bruce 2005; Frostegard 2005; Shoenfeld et al. 2005) and therefore, understanding the pathogenesis of this process seems important to give lupus patients a better long-term outcome and quality of life. Indeed, these patients are known to have an increased frequency of traditional risk factors such as hypertension, dyslipidemia, obesity and diabetes mellitus (Petri et al. 1992; Bruce et al. 1999; Svenungsson et al. 2001; Bruce 2005). However, it has now become clear that atherosclerosis cannot be entirely explained by Framingham risk factors and may be associated with a combination of both traditional and nontraditional risk factors (Esdaile et al. 2001; Svenungsson et al. 2001; Lee et al. 2006).

Interestingly, immune and inflammatory mechanisms seem to have a role in the pathogenesis of atherosclerotic vascular damage (Ross 1993; Ross 1999; Libby 2002). This raises the exciting possibility that SLE itself may be atherogenic through chronic activation of the immune system and inflammatory process. In fact, autoantibodies such as antiphospholipid, anti-β2-glycoprotein I, antioxidized low-density lipoprotein and anti-lipoprotein lipase and elevated inflammatory markers such as C reactive proteins (CRP) and interleukin 6 (IL-6) are common findings of SLE.

It is therefore reasonable to postulate that the interaction of disease associated factors induces specific alterations in lipoprotein metabolism which is aggravated by drugs and associated conditions frequently observed in lupus patients.

### The "lupus pattern" of dyslipoproteinemia

SLE is alleged as a classic model of a chronic immune complex-mediated inflammatory disease and probably several conditions detected in this disorder may enhance atherogenesis. Similarly, atherosclerosis is now assumed as a chronic process closely related to inflammation, which plays a pivotal role in the mechanisms of induction and progression of the atherosclerotic process in vessels (Ross 1993; Ross 1999; Libby 2002). It is well known that the acute-phase response promotes an altered hepatic synthesis

Correspondence: E. F. Borba, Faculdade de Medicina da USP, Reumatologia, Av. Dr. Arnaldo, 455-3° andar-Sala 3133, CEP: 01246-903, São Paulo, SP, Brazil. Tel/Fax: 55 11 30667490. E-mail: reumato@edu.usp.br

ISSN 1740-2522 print/ISSN 1740-2530 online © 2006 Taylor & Francis
DOI: 10.1080/17402520600876945

of a wide array of proteins involved in coagulation, in the complement system, and in lipoprotein metabolism (Gabay and Kushner 1999). Therefore, it seems reasonable to accept that inflammatory conditions of the disease itself would induce specific alterations in the lipid profile.

Reinforcing this possibility, sporadic cases of hyperlipoproteinemia type I (Glueck et al. 1969a,b; Pauciullo et al. 1986), and V (Alverson and Chase 1977) preceding SLE have been reported and provide support for the direct involvement of the disease in this process. Subsequently, definitive evidence for primary abnormalities of lipoprotein metabolism due to SLE *per se* was recognized (Ilowite et al. 1988; Borba and Bonfa 1997). The "lupus pattern" of dyslipoproteinemia identified was characterized by elevated levels of very low-density lipoprotein cholesterol (VLDL) and triglycerides (TG), and lower high-density lipoprotein cholesterol (HDL) levels (Ilowite et al. 1988; Borba and Bonfa 1997). Interestingly, activity seems to worsen this dyslipoproteinemia since a striking increase of VLDL and TG levels and decrease of HDL levels were directly correlated with SLEDAI scores (Borba and Bonfa 1997).

It should be emphasized that the most common abnormality in SLE is low HDL levels, which are detected in almost 80% of patients with active disease but also in approximately one-third of inactive patients (Borba and Bonfa 1997). Since HDL, levels are known to be inversely related to coronary artery disease (CAD) risk (Gordon et al. 1977; Castelli et al. 1986; Jacobs et al. 1990) this finding is certainly of concern. Noteworthy, an increase in triglyceride levels determined low HDL levels (Deckelbaum et al. 1984; Schaefer et al. 1987) and hypertriglyceridemia itself is also recognized as an independent risk factor in women (Castelli 1986; Castelli 1988; Bass et al. 1993). Together, these data suggest that SLE itself promotes a proatherogenic lipid profile.

These abnormalities observed in untreated active and inactive SLE might be explained by an accumulation of the main triglyceride-rich lipoproteins, namely chylomicrons and VLDL. In their catabolism, both lipoproteins undergo degradation by lipoprotein lipase (LPL) which is the enzyme involved in the lipolysis process (Goldberg 1996). Indeed, a low LPL activity results in accumulation of not only chylomicron but also VLDL, leading to high TG and low HDL levels (Deckelbaum et al. 1984; Schaefer et al. 1987).

Interestingly, a significant impairment of LPL activity in SLE was observed using an *in vitro* lipolysis assay (Borba et al. 2000). Moreover, a striking reduction of the intravascular lipolysis of chylomicron by LPL associated with a pronounced delay in removal of their remnants by liver receptors was also demonstrated in SLE patients (Borba et al. 2000). Furthermore, the recent description of high frequency of antibodies to lipoprotein lipase (anti-LPL) in SLE raises the possibility that these autoantibodies may modulate LPL activity in SLE (Reichlin et al. 2002; de Carvalho et al. 2004) as has been described for autoimmune hyperlipidemia (Beaumont and Beaumont 1977).

Remarkably, the presence of these autoantibodies was not only significantly associated with increased triglyceride levels (Reichlin et al. 2002; de Carvalho et al. 2004) but also with disease activity (SLEDAI), ESR and CRP (de Carvalho et al. 2004), suggesting that immune mechanism and inflammatory process are concomitantly involved in SLE dyslipoproteinemia.

### Cytokines and lupus dyslipoproteinemia

The possible role of inflammation in modulating LPL enzyme is emphasized by the recent description of a significant down-regulation of LPL activity induced by TNF, IL-1 and IFN-gamma (Enholm et al. 1982; Beutler and Cerami 1985; Semb et al. 1987). An enhanced production of these cytokines is a characteristic of SLE, particularly during active disease (Hooks et al. 1982; Tanaka et al. 1988; Maury and Teppo 1989; Spronk et al. 1992).

In this regard, it is known that tumor necrosis factor (TNF) promotes a prompt increase in circulating TG when administered to humans (Feinberg et al. 1988) which is due to an increase hepatic synthesis of VLDL. Moreover, TNF have the ability to inhibit LPL (Beutler et al. 1985). It was recently described that levels of circulating TNF are raised in SLE and correlate with active disease and TG levels (Svenungsson et al. 2003a,b). In addition, TNF activity was closely associated with elevated TGs among SLE patients with previous cardiovascular disease (Svenungsson et al. 2003a). Interestingly, HDL-associated apolipoprotein A-I has been shown to decrease TNF production through its inhibition of contact-mediated activation of monocytes by binding to stimulated T cells (Hyka et al. 2001). In this regard, low HDL levels seem to be not only a consequence of active disease but may also indirectly contribute to enhance inflammation in SLE.

Additionally, in SLE the altered lipid profile may be influenced by high concentrations of two other proatherogenic cytokines: monocyte chemoattractant protein-1 (MCP-1) and IL-6 (Asanuma et al. 2006). In fact, MCP-1 concentrations are related with monocyte infiltration in atherosclerotic lesions and are associated with atherosclerosis in the general population. Moreover, it has been demonstrated that anti-MCP-1 gene therapy limited progression and destabilization of atherosclerosis in animal models (Reckless et al. 1999; Inoue et al. 2002). Interestingly, higher MCP-1 levels were detected in SLE and were particularly related with disease activity and with increased TG levels (Asanuma et al. 2006). Furthermore, high IL-6 concentrations were associated with low HDL levels (Asanuma et al. 2006) in the same study reinforcing the relevance of cytokines in the lipid profile of SLE patients.

Accordingly, TNF-alpha and interleukins-1 and 6 are known to stimulate the CRP synthesis by the liver (Gabay and Kushner 1999). The additional observation that these cytokines can affect LPL function (Beutler and Cerami 1985; Semb et al. 1987) further supports their role in lupus dyslipoproteinemia. CRP levels are also increased in the metabolic syndrome (Festa et al. 2000; Tamakoshi et al. 2003; Aronson et al. 2004), hypertriglyceridemia (Grau et al. 1996; Tamakoshi et al. 2003; Fredrikson et al. 2004; Jialal et al. 2004) and in patients with low levels of HDL cholesterol (Tamakoshi et al. 2003; Fredrikson et al. 2004; Jialal et al. 2004).

### Other autoantibodies and lupus dyslipoproteinemia

It has been extensively demonstrated that hypertriglyceridemia enhances replacement of cholesterol esters in the core of LDL particles, creating small dense LDL (Richards et al. 1989) which are easily filtered into the arterial wall (Stender and Zilversmit 1981) and more susceptible to oxidation (Chait et al. 1993). Oxidized LDL (oxLDL) may lead to the formation of antigenic neoepitopes that bind to scavenger receptors of macrophages, enhancing oxLDL uptake with subsequent foam cell formation (Heinecke 1997). On the other hand, HDL has an antiatherogenic effect by inhibiting LDL oxidation (Parthasarathy et al. 1990), reducing LDL lipid hydroperoxides (Van Lenten et al. 1995) and oxidized phospholipids (Navab et al. 2004), and transporting oxidized lipids to the liver in the reverse cholesterol transport (Fluiter et al. 1996).

Therefore, the "lupus pattern" of dyslipoproteinemia characterized by hypertriglyceridemia and low HDL should certainly contribute for an enhanced LDL oxidation (oxLDL). Indeed, high levels of oxLDL (i.e. Apo B lipoproteins containing oxPL) were recently identified in this disease (Frostegard et al. 2005). Furthermore, antibodies to oxLDL epitopes were described in SLE (Vaarala et al. 1993; George et al. 1999; Wu et al. 1999; Frostegard et al. 2005). It also seems possible that these antibodies would enhance the accumulation of oxidized LDL to the endothelial wall by increasing LDL uptake via Fc-receptor, as first hypothesized by Vaarala et al. (1993). Of interest, a possible crossreaction between anti-oxLDL and cardiolipin antibodies was also reported in SLE (Vaarala et al. 1993; George et al. 1999; Wu et al. 1999; Frostegard et al. 2005), as well as a cross-reactivity between aCL, anti-HDL and anti-apoA1 IgG antibodies (Delgado et al. 2003). These findings may explain in part the association of IgG anticardiopilin (aCL) with low HDL and low apolipoprotein A-1 (apoA1) levels in these patients (Lahita et al. 1993). Intriguingly, high levels of anti-apoA1 antibodies were associated to low HDL levels in SLE (Dinu et al. 1998a,b) and further studies are needed to clarify its participation in the HDL metabolism.

High serum levels of oxLDL/β2GPI complexes and IgG antibodies to these complexes were also detected in SLE (Kobayashi et al. 2001, 2003; Lopez et al. 2005, 2006). These atherogenic complexes are formed mainly in the atherosclerotic lesions and thus indicate a widespread underlying oxidative injury (Lopez et al. 2006). β2GPI seems to bind to oxLDL to counteract its inflammatory effect (oxidative) and to promote macrophage uptake via scavenger but the large complexes are usually deposited and promote foam cells formation. In addition, the presence of anti-β2GPI antibodies enhances *in vitro* macrophage uptake of these complexes (Kobayashi et al. 2001). Interestingly, oxLDL/β2GPI complexes and IgG anti-/β2GPI antibodies were detected in lupus patients that had high TG and low HDL levels (Lopez et al. 2006).

### Drugs in lupus dyslipoproteinemia

Several drugs commonly used in the treatment of SLE also induce undesirable effects on lipids but corticosteroids are always remembered since their alterations were extensively studied in SLE (Ettinger et al. 1987; Ettinger and Hazzard 1988; Ilowite et al. 1988; MacGregor et al. 1992; Leong et al. 1994; Petri et al. 1994; Bruce et al. 1999).

In fact, chronic corticosteroids use in SLE is associated to increase total plasma cholesterol and its fractions (LDL and HDL) levels, and also TG (Ettinger et al. 1987; Ettinger and Hazzard 1988; Ilowite et al. 1988; MacGregor et al. 1992; Leong et al. 1994; Petri et al. 1994; Bruce et al. 1999), which seem mediated by increased plasma insulin levels and lipid production by the liver, and impaired lipid catabolism (Sholter and Armstrong 2000). This effect could be identified after a short period of 1–2 months of this therapy (Ilowite et al. 1988) and is also dose related since low prednisone daily doses do not significantly alter the lipid profile (MacGregor et al. 1992; Petri et al. 1994). In fact, Petri et al showed that for each 10 mg increase in prednisone dosage, there is a 7.5 mg% corresponding increase in serum cholesterol and a 1.1 mm Hg increase in mean arterial pressure (Petri et al. 1994). In addition, corticosteroids can also indirectly promote other traditional risk factors such as obesity, systemic hypertension, glucose intolerance and diabetes (Petri et al. 1992). Interestingly, Manzi et al found that corticosteroid use was associated with myocardial infarct (Manzi et al. 1997) and carotid atherosclerosis in SLE patients, independent of the development or worsening of hypertension and diabetes (Manzi et al. 1999).

On the other hand, a possible antihyperlipidemic property of antimalarials in SLE was first demonstrated in a combined group of patients with rheumatoid arthritis and SLE treated with hydroxychloroquine (Wallace et al. 1990). Subsequently, Petri et al found that the use of hydroxychloroquine was associated with an 8.5% decrease in serum cholesterol (Petri et al. 1994). Thereafter, studies that specifically addressed its effect on lipoproteins in lupus patients had confirmed the beneficial effect on lipids (Hodis et al. 1993; Kavanaugh et al. 1997; Rahman et al. 1999; Borba and Bonfa 2001). Interestingly, a significant increase in HDL levels is observed in SLE patients taking antimalarials with or without prednisone (Borba and Bonfa 2001). Moreover, a beneficial effect in the diabetes control has been suggested in randomized trials after the use of hydroxychloroquine probably by the alteration of insulin resistance (Powrie et al. 1993; Gerstein et al. 2002).

Importantly, several other drugs such as anticonvulsants, antihypertensives (beta blockers and diuretics), estrogen and/or progesterone containing agents should also be considered since they can promote specific alterations on the lipid profile (Henkin et al. 1992).

### Associated diseases in lupus dyslipoproteinemia

Renal involvement, such as uremia and nephrotic syndrome, is frequently observed in the course of SLE, and theses conditions are known to alter the lipid profile (Appel et al. 1985; Attman and Alaupovic 1991). Also to be considered is the concurrence of other systemic disorders such as thyroid disease and diabetes mellitus, which promote distinct changes in lipoprotein metabolism (Thompson et al. 1981; Brown 1994). Additionally, as patients live longer, lipid abnormalities due to increasing age (Heiss et al. 1980) and menopause (Kannel et al. 1976) may become relevant.

### References

Alverson DC, Chase HP. 1977. Systemic lupus erythematosus in childhood presenting as hyperlipoproteinemia. J Pediatr 91:72–75.

Appel GB, Blum CB, Chien S, et al. 1985. The hyperlipidemia of the nephrotic syndrome. Relation to plasma albumin concentration, oncotic pressure, and viscosity. N Engl J Med 312(24):1544–1548.

Aronson D, Sella R, Sheikh-Ahmad M, et al. 2004. The association between cardiorespiratory fitness and C-reactive protein in subjects with the metabolic syndrome. J Am Coll Cardiol 44: 2003–2007.

Asanuma Y, Chung CP, Oeser A, et al. 2006. Increased concentration of proatherogenic inflammatory cytokines in systemic lupus erythematosus: Relationship to cardiovascular risk factors. J Rheumatol 33(3):539–545.

Attman PO, Alaupovic P. 1991. Lipid and apolipoprotein profiles of uremic dyslipoproteinemia—relation to renal function and dialysis. Nephron 57(4):401–410.

Bass KM, Newschaffer CJ, Klag MJ, Bush TL. 1993. Plasma lipoprotein levels as predictors of cardiovascular death in women. Arch Intern Med 153:2209–2216.

Beaumont JL, Beaumont V. 1977. Autoimmune hyperlipidemia. Atherosclerosis 26:405–418.

Beutler BA, Cerami A. 1985. Recombinant interleukin 1 suppresses lipoprotein lipase activity in 3T3-L1 cells. J Immunol 135: 3969–3971.

Beutler B, Mahoney J, Le Trang N, et al. 1985. Purification of cachectin, a lipoprotein lipase-suppressing hormone secreted by endotoxin-induced RAW 264.7 cells. J Exp Med 161:984–995.

Borba EF, Bonfa E. 1997. Dyslipoproteinemias in systemic lupus erythematosus: Influence of disease, activity, and anticardiolipin antibodies. Lupus 6(6):533–539.

Borba EF, Bonfa E. 2001. Longterm beneficial effect of chloroquine diphosphate on lipoprotein profile in lupus patients with and without steroid therapy. J Rheumatol 28:780–785.

Borba EF, Bonfa E, Vinagre CG, et al. 2000. Chylomicron metabolism is markedly altered in systemic lupus erythematosus. Arthritis Rheum 43:1033–1040.

Brown WV. 1994. Lipoprotein disorders in diabetes mellitus. Med Clin North Am 78:143–161.

Bruce IN. 2005. 'Not only…but also': Factors that contribute to accelerated atherosclerosis and premature coronary heart disease in systemic lupus erythematosus. Rheumatology (Oxford) 44(12):1492–1502.

Bruce IN, Urowitz MB, Gladman DD, et al. 1999. Natural history of hypercholesterolemia in systemic lupus erythematosus. J Rheumatol 26:2137–2143.

de Carvalho JF, Borba EF, Viana VS, et al. 2004. Anti-lipoprotein lipase antibodies: A new player in the complex atherosclerotic process in systemic lupus erythematosus? Arthritis Rheum 50(11):3610–3615.

Castelli WP, Garrison RJ, Wilson PW, et al. 1986. Incidence of coronary heart disease and lipoprotein cholesterol levels. The Framingham study. JAMA 256:2835–2838.

Castelli WP. 1988. Cardiovascular disease in women. Am J Obstet Gynecol 158:1553–1560.

Castelli WP. 1986. The triglyceride issue: A view from Framingham. Am Heart J 112:432–437.

Chait A, Brazg RL, Tribble DL, et al. 1993. Susceptibility of small, dense, low-density lipoproteins to oxidative modification in subjects with the atherogenic lipoprotein phenotype, pattern B. Am J Med 94:350–356.

Deckelbaum RJ, Granot E, Oschry Y, et al. 1984. Plasma triglyceride determines structure-composition in low and high density lipoproteins. Arteriosclerosis 4:225–231.

Delgado AJ, Kumar S, Isenberg DA. 2003. Cross-reactivity between anti-cardiolipin, anti-high-density lipoprotein and anti-apolipoprotein A-I IgG antibodies in patients with systemic lupus erythematosus and primary antiphospholipid syndrome. Rheumatology 42:893–899.

Dinu AR, Merrill JT, Shen C, et al. 1998a. Frequency of antibodies to the cholesterol transport protein apolipoprotein A1 in patients with SLE. Lupus 7:355–360.

Dinu AR, Merrill JT, Sutton-Tyrell K, et al. 1998b. Autoantibodies to apolipoprotein A1 (APOA1) in an SLE population: Relationship to HDL levels and to carotid atherosclerosis by ultrasound assessment. Arthritis Rheum 41(Suppl):S139.

Enholm C, Aho K, Huttunen JK, et al. 1982. Effect of interferon on plasma lipoproteins and on the activity of postheparin lipases. Arteriosclerosis 2(1):69–73.

Esdaile JM, Abrahamowicz M, Grodzicky T, et al. 2001. Traditional Framingham risk factors fail to fully account for accelerated atherosclerosis in systemic lupus erythematosus. Arthritis Rheum 44:2331–2337.

Ettinger WH, Hazzard WR. 1988. Elevated apolipoprotein-B levels in corticosteroid-treated patients with systemic lupus erythematosus. J Clin Endocrinol Metab 67:425–428.

Ettinger WH, Goldberg AP, Applebaum-Bowden D, Hazzard WR. 1987. Dyslipoproteinemia in systemic lupus erythematosus. Effects of corticosteroids. Am J Med 83:503–508.

Feinberg B, Kurzrock R, Talpaz M, et al. 1988. A phase I trial of intravenously-administered recombinant tumor necrosis factor-alpha in cancer patients. J Clin Oncol 6(8):1328–1334.

Festa A, D'Agostino R Jr, Howard G, et al. 2000. Chronic subclinical inflammation as part of the insulin resistance syndrome: The insulin resistance atherosclerosis study (IRAS). Circulation 102:42–47.

Fluiter K, Vietsch H, Biessen EA, et al. 1996. Increased selective uptake *in vivo* and *in vitro* of oxidized cholesteryl esters from high-density lipoprotein by rat liver parenchymal cells. Biochem J 319:471–476.

Fredrikson GN, Hedblad B, Nilsson JA, et al. 2004. Association between diet, lifestyle, metabolic cardiovascular risk factors, and plasma C-reactive protein levels. Metabolism 53:1436–1442.

Frostegard J. 2005. SLE, atherosclerosis and cardiovascular disease. J Intern Med 257(6):485–495.

Frostegard J, Svenungsson E, Wu R, et al. 2005. Lipid peroxidation is enhanced in patients with systemic lupus erythematosus and is associated with arterial and renal disease manifestations. Arthritis Rheum 52(1):192–200.

Gabay C, Kushner I. 1999. Acute-phase proteins and other systemic responses to inflammation. N Engl J Med 340(6):448–454.

George J, Harats D, Gilburd B, et al. 1999. Atherosclerosis-related markers in systemic lupus erythematosus patients: The role of humoral immunity in enhanced atherogenesis. Lupus 8: 220–226.

Gerstein HC, Thorpe KE, Taylor DW, Haynes RB. 2002. The effectiveness of hydroxychloroquine in patients with type 2 diabetes mellitus who are refractory to sulfonylureas—a randomized trial. Diabetes Res Clin Pract 55:209–219.

Glueck CJ, Levy RI, Glueck HI, et al. 1969a. Acquired type I hyperlipoproteinemia with systemic lupus erythematosus, dysglobulinemia and heparin resistance. Am J Med 47(2): 318–324.

Glueck CJ, Kaplan AP, Levy RI, et al. 1969b. A new mechanism of exogenous hyperglyceridemia. Ann Intern Med 71(6): 1051–1062.

Goldberg IJ. 1996. Lipoprotein lipase and lipolysis: Central roles in lipoprotein metabolism and atherogenesis. J Lipid Res 37:693–707.

Gordon T, Castelli WP, Hjortland MC, et al. 1977. High density lipoprotein as a protective factor against coronary heart disease. Am J Med 62:707–714.

Grau AJ, Buggle F, Becher H, et al. 1996. The association of leukocyte count, fibrinogen and C-reactive protein with vascular risk factors and ischemic vascular diseases. Thromb Res 82:245–255.

Hahn BH. 2003. Systemic lupus erythematosus and accelerated atherosclerosis. N Engl J Med 349(25):2379–2380.

Heinecke JW. 1997. Mechanisms of oxidative damage of low-density lipoprotein in human atherosclerosis. Curr Opin Lipidol 8:268–274.

Heiss G, Tamir I, Davis CE, et al. 1980. Lipoprotein-cholesterol distribution in selected North American populations: The lipid research clinics program prevalence study. Circulation 61:302–315.

Henkin Y, Como JA, Oberman A. 1992. Secondary dyslipidemia. Inadvertent effects of drugs in clinical practice. JAMA 267:961–968.

Hodis HN, Quismorio FP, Wickham E, Blankenhorn DH. 1993. The lipid, lipoprotein, and apolipoprotein effects of hydroxychloroquine in patients with systemic lupus erythematosus. J Rheumatol 20:661–665.

Hooks JJ, Jordan GW, Cupps T, et al. 1982. Multiple interferons in the circulation of patients with systemic lupus erythematosus and vasculitis. Arthritis Rheum 25:396–400.

Hyka N, Dayer JM, Modoux C, et al. 2001. Apolipoprotein A-I inhibits the production of interleukin-1_ and tumor necrosis factor-_ by blocking contact-mediated activation of monocytes by T lymphocytes. Blood 97:2381–2389.

Ilowite NT, Samuel P, Ginzler E, et al. 1988. Dyslipoproteinemia in pediatric systemic lupus erythematosus. Arthritis Rheum 31:859–863.

Inoue S, Egashira K, Ni W, et al. 2002. Anti-monocyte chemoattractant protein-1 gene therapy limits progression and destabilization of establish atherosclerosis in apolipoprotein E-knockout mice. Circulation 106:2700–2706.

Jacobs DR Jr, Mebane IL, Bangdiwala SI, et al. 1990. High density lipoprotein cholesterol as a predictor of cardiovascular disease mortality in men and women: The follow-up study of the lipid research clinics' prevalence study. Am J Epidemiol 131:32–47.

Jialal I, Devaraj S, Venugopal SK. 2004. C-reactive protein: Risk marker or mediator in atherothrombosis? Hypertension 44:6–11.

Kannel WB, Hjortland MC, McNamara PM, et al. 1976. Menopause and risk of cardiovascular disease. The Framingham study. Ann Intern Med 85:447–452.

Kavanaugh A, Adams-Huet B, Jain R, Denke M, McFarlin J. 1997. Hydroxychloroquine effects on lipoprotein profiles (the HELP trial): A double-blind, randomized, placebo-controlled, pilot study in patients with systemic lupus erythematosus. J Clin Rheumatol 3:3–8.

Kobayashi K, Matsuura E, Liu Q, et al. 2001. A specific ligand for beta(2)-glycoprotein I mediates autoantibody-dependent uptake of oxidized low density lipoprotein by macrophages. J Lipid Res 42(5):697–709.

Kobayashi K, Kishi M, Atsumi T, et al. 2003. Circulating oxidized LDL forms complexes with beta2-glycoprotein I: Implication as an atherogenic autoantigen. J Lipid Res 44(4):716–726.

Lahita RG, Rivkin E, Cavanagh I, et al. 1993. Low levels of total cholesterol, high-density lipoprotein, and apolipoprotein A1 in association with anticardiolipin antibodies in patients with systemic lupus erythematosus. Arthritis Rheum 36:1566–1574.

Lee AB, Godfrey T, Rowley KG, et al. 2006. Traditional risk factor assessment does not capture the extent of cardiovascular risk in systemic lupus erythematosus. Intern Med J 36(4):237–243.

Leong KH, Koh ET, Feng PH, Boey ML. 1994. Lipid profiles in patients with systemic lupus erythematosus. J Rheumatol 21:1264–1267.

Libby P. 2002. Inflammation in atherosclerosis. Nature 420(6917):868–874, 19–26 December.

Lopez LR, Simpson DF, Hurley BL, et al. 2005. OxLDL/{beta} 2GPI complexes and autoantibodies in patients with systemic lupus erythematosus, systemic sclerosis, and antiphospholipid syndrome: Pathogenic implications for vascular involvement. Ann N Y Acad Sci 1051:313–322.

Lopez LR, Salazar-Paramo M, Palafox-Sanchez C, et al. 2006. Oxidized low-density lipoprotein and beta2-glycoprotein I in patients with systemic lupus erythematosus and increased carotid intima-media thickness: Implications in autoimmune-mediated atherosclerosis. Lupus 15(2):80–86.

MacGregor AJ, Dhillon VB, Binder A, et al. 1992. Fasting lipids and anticardiolipin antibodies as risk factors for vascular disease in systemic lupus erythematosus. Ann Rheum Dis 51:152–155.

Manzi S, Meilahn EN, Rairie J, et al. 1997. Age-specific incidence rates of myocardial infarction and angina in women with systemic lupus erythematosus: Comparison with the Framingham study. Am J Epidemiol 145:408–415.

Manzi S, Selzer F, Sutton-Tyrrel K, et al. 1999. Prevalence and risk factors of carotid plaque in women with systemic lupus erythematosus. Arthritis Rheum 42:51–60.
Maury CPJ, Teppo AM. 1989. Tumor necrosis factor in the serum of patients with systemic lupus erythematosus. Arthritis Rheum 32:146–150.
Navab M, Ananthramaiah GM, Reddy ST, et al. 2004. The oxidation hypothesis of atherogenesis: The role of oxidized phospholipids and HDL. J Lipid Res 45:993–1007.
Parthasarathy S, Barnett J, Fong LG. 1990. High-density lipoprotein inhibits the oxidative modification of low-density lipoprotein. Biochim Biophys Acta 1044:275–283.
Pauciullo P, De Simone B, Rubba P, et al. 1986. A case of association between type I hyperlipoproteinemia and systemic lupus erythematosus (SLE). Effects of steroid treatment. J Endocrinol Invest 9:517–520.
Petri M, Perez-Gutthann S, Spence D, et al. 1992. Risk factors for coronary artery disease in patients with systemic lupus erythematosus. Am J Med 93:513–519.
Petri M, Lakata C, Magder L, Goldman D. 1994. Effect of prednisone and hydroxychloroquine on coronary artery disease risk factors in systemic lupus erythematosus: A longitudinal data analysis. Am J Med 96:254–259.
Powrie JK, Shojaee-Moradie F, Watts GF, et al. 1993. Effects of chloroquine on the dyslipidemia of non-insulin-dependent diabetes mellitus. Metabolism 42:415–419.
Rahman P, Gladman DD, Urowitz MB, et al. 1999. The cholesterol lowering effect of antimalarial drugs is enhanced in patients with lupus taking corticosteroid drugs. J Rheumatol 26:325–330.
Reckless J, Rubin EM, Verstuyft JB, et al. 1999. Monocyte chemoattractant protein-1 but not tumor necrosis factor-alpha is correlated with monocyte infiltration in mouse lipid lesions. Circulation 99:2310–2316.
Reichlin M, Fesmire J, Quintero-Del-Rio AI, et al. 2002. Autoantibodies to lipoprotein lipase and dyslipidemia in systemic lupus erythematosus. Arthritis Rheum 46(11): 2957–2963.
Richards GE, Grundy SM, Cooper K. 1989. Influence of plasma triglycerides on lipoprotein patterns in normal subjects and in patients with coronary artery disease. Am J Cardiol 63:1214–1220.
Ross R. 1993. The pathogenesis of atherosclerosis: A perspective for the 1990s. Nature 362(6423):801–809.
Ross R. 1999. Atherosclerosis—an inflammatory disease. N Engl J Med 340(2):115–126.
Schaefer EJ, et al. 1987. Plasma triglycerides in regulation of HDL-cholesterol levels. Lancet ii:391–392.
Semb H, Peterson J, Tavernier J, et al. 1987. Multiple effects of tumor necrosis factor on lipoprotein lipase *in vivo.* J Biol Chem 262:8390–8394.
Shoenfeld Y, Gerli R, Doria A, et al. 2005. Accelerated atherosclerosis in autoimmune rheumatic diseases. Circulation 112(21):3337–3347.
Sholter DE, Armstrong PW. 2000. Adverse effects of corticosteroids on the cardiovascular system. Can J Cardiol 16:505–511.
Spronk PE, Ter Borg EJ, Limburg PC, et al. 1992. Plasma concentration of IL-6 in systemic lupus erythematosus; an indicator of disease activity? Clin Exp Immunol 90:106–110.
Stender S, Zilversmit DB. 1981. Transfer of plasma lipoprotein components and plasma proteins into aortas of cholesterol-fed rabbits: Molecular size as a determinant of plasma lipoprotein influx. Arteriosclerosis 1:28–49.
Svenungsson E, Jensen-Urstad K, Heimburger M, et al. 2001. Risk factors for cardiovascular disease in systemic lupus erythematosus. Circulation 104:1887–1893.
Svenungsson E, Fei GZ, Jensen-Urstad K, et al. 2003a. TNF-alpha: A link between hypertriglyceridaemia and inflammation in SLE patients with cardiovascular disease. Lupus 12(6):454–461.
Svenungsson E, Gunnarsson I, Fei GZ, et al. 2003b. Elevated triglycerides and low levels of high-density lipoprotein as markers of disease activity in association with up-regulation of the tumor necrosis factor alpha/tumor necrosis factor receptor system in systemic lupus erythematosus. Arthritis Rheum 48(9):2533–2540.
Tamakoshi K, Yatsuya H, Kondo T, et al. 2003. The metabolic syndrome is associated with elevated circulating C-reactive protein in healthy reference range, a systemic low-grade inflammatory state. Int J Obes Relat Metab Disord 27:443–449.
Tanaka Y, Saito K, Shirakawa F, et al. 1988. Production of B cell-stimulating factors by B cells in patients of systemic lupus erythematosus. J Immunol 141:3043–3049.
Thompson GR, Soutar AK, Spengel FA, et al. 1981. Defects of receptor-mediated low density lipoprotein catabolism in homozygous familial hypercholesterolemia and hypothyroidism *in vivo.* Proc Natl Acad Sci USA 78(4):2591–2595.
Vaarala O, Alfthan G, Jauhiainen M, et al. 1993. Crossreaction between antibodies to oxidised low density lipoprotein and to cardiolipin in systemic lupus erythematosus. Lancet 341: 923–925.
Van Lenten BJ, Hama SY, de Beer FC, et al. 1995. Anti-inflammatory HDL becomes pro-inflammatory during the acute phase response. Loss of protective effect of HDL against LDL oxidation in aortic wall cell cocultures. J Clin Invest 96:2758–2767.
Wallace DJ, Metzger AL, Stecher VJ, Tumbull BA, Kern PA. 1990. Cholesterol-lowering effect of hydroxychloroquine in patients with rheumatic disease: Reversal of deleterious effects of steroids on lipids. Am J Med 89:322–326.
Wu R, Svenungsson E, Gunnarsson I, et al. 1999. Antibodies against lysophosphatidylcholine and oxidized LDL in patients with SLE. Lupus 8:142–150.

